# Prevalence of binary-toxin genes (*cdtA* and *cdtB*) among clinical strains of *Clostridium difficile* isolated from diarrheal patients in Iran 

**Published:** 2018

**Authors:** Masoumeh Azimirad, Fatemeh Naderi Noukabadi, Farhad Lahmi, Abbas Yadegar4

**Affiliations:** 1 *Gastroenterology and Liver Diseases Research Center, Research Institute for Gastroenterology and Liver Diseases, Shahid Beheshti University of Medical Sciences, Tehran, Iran.*; 2 *Basic and Molecular Epidemiology of Gastrointestinal Disorders Research Center, Research Institute for Gastroenterology and Liver Diseases, Shahid Beheshti University of Medical Sciences, Tehran, Iran.*; 3 *AJA Cancer Epidemiology Research and Treatment Center (AJA- CERTC), AJA University of Medical Sciences, Tehran, Iran*; 4 *Foodborne and Waterborne Diseases Research Center, Research Institute for Gastroenterology and Liver Diseases, Shahid Beheshti University of Medical Sciences, Tehran, Iran*

**Keywords:** *Clostridium difficile*, Binary toxin, *cdtA*, *cdtB*, Diarrheal patients

## Abstract

**Aim:**

In this study we investigated the prevalence of binary toxin genes, *cdtA* and *cdtB*, in clinical isolates of *C. difficile* from hospitalized patients with diarrhea.

**Background:**

*C. difficile* binary toxin (CDT) is an action-specific ADP-ribosyltransferase that is produced by some strains of *C. difficile*. Co-expression of this toxin with *tcdA* and *tcdB* can lead to more severe disease in CDI patients.

**Methods:**

Totally, 930 patients suspected of having CDI was included in this study. All samples were treated with methanol and cultured on selective C. difficile agar plates. The *C. difficile* isolates were further identified by PCR. Presence of *tcdA*, *tcdB*, *cdtA*, and *cdtB* genes among the strains were examined by PCR.

**Results:**

Analysis of the PCR results showed a prevalence of 85.2% (144/169) for toxigenic *C. diffidile*. Toxin genotyping of the strains for *tcdA* and *tcdB* genes revealed the toxin profiles of A+B+, A+B-, A-B+ accounting for 86.1% (124/144), 7.6% (11/144), 6.2% (9/144) among the strains, respectively. Totally, 12.4% (21/169) of the *C. difficile* strains were binary toxin-positive. *cdtA*-B+, *cdtA*+‏B‏+ and *cdtA*+B- were detected in 43% (9/21), 38% (8/21) and 19% (4/21) of the strains, respectively. Interestingly, 12% (3/25) of nontoxigenic *C. difficile* strains (*tcdA*-B-) had either *cdtA*+‏B‏+ or *cdtA*-B+ profiles.

**Conclusion:**

This is the first report for the prevalence of binary toxin genes in *C. difficile* strains isolated from Iran. Further studies are required to investigate the exact role of binary toxins in the pathogenesis of *C. difficile* particularly in patients with chronic diarrhea among Iranian populations.

## Introduction

*Clostridium difficile *is a gram-positive spore forming anaerobic bacterial pathogen. This microorganism is considered as the major causative agent for 5 to 25% of antibiotic associated diarrhea (AAD) and also pseudomembranous colitis (PMC) (1). Toxigenic strains generally produce two main toxins, including *toxin A* (enterotoxin) and *B* (cytotoxin), which have been identified as the main virulence factors in the pathogenesis of these bacteria (2, 3). The genes encoding *toxin A* and *B *are clustered in a specific locus, the pathogenicity locus called PaLoc. This locus is composed of five genes, including *tcdA* and *tcdB* that encode the over-mentioned toxins, *tcdE* encodes a putative holin for extracellular release of *tcdA* and *tcdB*, and *tcdR* and *tcdC* that encode the regulatory proteins (4).

Popoff *et al.* discovered a binary toxin in the historic CD196 strain, called CDT in 1988 (5). This toxin is found in a few strains of *C. difficile* and consists of a binding component and an enzymatic component that displays an action-specific ADP ribosyltransferase activity that leads to cytoskeleton disorganization (6). The genes encoding these two components, *cdtA* and *cdtB*, and a regulatory protein, are co-located on a locus called CdT. This toxin might potentiate the toxicity of *tcdA* and *tcdB* and lead to more severe disease and could, thus, be considered to be an accessory virulence factor (7). It has been estimated that about 1.6 to 10% of *C. difficile* isolates carry the binary toxin genes in their genomes (8).

In spite of high prevalence of toxin-A-negative/toxin-B-positive *C. difficile* strains among hospitalized patients, there are several reports about involvement of *tcdA/B*-negative strains in the occurrence of AAD and enterocolitis (9-13). Although overgrowth of *tcdA/B*-negative strains in the intestine and the absence of other enteric pathogens may support this involvement, further studies are required to determine the role of accessory virulence factors in the pathogenesis of *C. difficile *infection (CDI). Involvement of CDT in induction of apoptosis through its DNAse activity was shown in an *in vitro* study (14). This activity could mimic the same pathophysiological effects in the intestine, as was shown for *tcdA/B* toxins (8). Several clinical studies have suggested an association between the CDT-encoding *C. difficile* strains and increased mortality of the patients (15), however its establishment needs further studies. In the current study we aimed to investigate the prevalence of binary toxin genes, *cdtA* and *cdtB*, in clinical isolates of *C. difficile* recovered from hospitalized patients with diarrhea. 

## Methods

**Bacterial isolates**

The study population consisted of 169 clinical isolates of *C. difficile,* which had been recovered from the fecal samples of 930 patients with chronic diarrhea referred to the Diagnostic Anaerobic Laboratory of Research Institute for Gastroenterology and Liver Diseases in Tehran, Iran. All demographic and clinical data of the patients, including age, gender, antibiotic treatment and medications history were collected by using a standard questionnaire.

**Culture and isolation**

All fecal samples from patients cultured on proper culture media after their treatments by the following methods. First, small amount of stool samples were mixed with 1 ml of 5% yeast extract broth and directly inoculated onto *C. difficile* medium (Mast, London, United Kingdom) supplemented with 7% horse blood and *C. difficile* selective supplement consisting of D-cycloserine (250 mg/ liter), cefoxitin (8 mg/liter), and lysozyme (5 mg/liter) (Mast, London, United Kingdom). The cultured plates were incubated at 37 °C for at least 48-72 h under anaerobic conditions (80% N_2_; 10% CO_2_ and 10% H_2_) in an anaerobic generation system Anoxomat-Mart (Microbiology, Holland). Second, the samples treated with 1 ml of methanol (alcohol-shock procedure) for 1 to 2 minutes before inoculation on *C. difficile* medium (16, 17). The cultures results were followed up to one week. Isolates with characteristic colony morphologies and Gram staining for *C. difficile* were further identified by PCR using specific primers (18). Subcultures of the confirmed isolates were stored in cooked meat broth (Himedia, India) at 4 ˚C.

**Genomic DNA extraction**

The genomic DNA was extracted from pure colonies of *C. difficile *grown on *C. difficile* medium agar plates. DNA was extracted by Instagene matrix extraction kit (Bio-Rad, Nazareth, Belgium) and boiling method (19). In the boiling method, a loop full of each colony was resolved in 500 µl of distilled water and homogenized and centrifuged for 10 min at 13000 g. The pellets were mixed with 100 µl of distilled water, vortexed and incubated for 10 min in water bath. The samples were then centrifuged at 13000 g for 8-10 min. The supernatant containing bacterial DNA was stored at -20 °C, until further use.

**Detection of binary-toxin genes**

For molecular identification of *C. difficile* isolates, PCR was done using specific primers for *cdd3 *gene of PaLoc. All primer pairs used in this study are presented in Table 1. PCR amplification was also done by using specific primers for *tcdA* and* tcdB* genes as previously described by Spigaglia *et al.* (18). For detection of *cdtA* and *cdtB* genes, PCR was done using specific primers (20) in a 25μl reaction mixtures containing 1X PCR buffer (50 mM KCl, 10 mM Tris-HCl), 1.5μM MgCl_2_, 0.3μM of *cdtA* and 0.5μM *cdtB* primers, and 1U of Taq DNA polymerase. PCR amplifications of 375 bp fragment of the *cdtA* was done in a thermocycler (Eppendorf, Hamburg, Germany) as follows: initial denaturation at 5 min at 95 °C, followed by 40 cycles of 1 min at 95 °C, 50s at 57.5 °C and 35s at 72 °C; and a final extension at 72 °C for 5 min to end amplification process. For amplification of 510 bp fragment of the* cdtB* the following time-temperature profile was used: 5 min at 95 °C for initial denaturation, 40 cycles of 1 min at 95 °C, 1 min at 58.9 °C, and 35 at 72 °C; and a final extension cycle of 10 min at 72 °C. *Peptoclostridium difficile* strain RIGLD141 was used as the control positive strain in amplification experiments. The partial nucleotide sequences for *cdtB* (KM047900.1) and *cdtA*-like (KM047901.1) genes were deposited in the GenBank/NCBI.

**Statistical analysis**

Statistical analyses were performed using IBM SPSS Statistics version 21 (Armonk, NY: IBM Corp.). Clinical and demographic data were analyzed by the Chi-square and Fisher’s exact tests. A *p* value less than 0.05 was regarded as indicating a statistically significant difference.

**Table 1:** Oligonucleotide sequences used to amplify the target genes in this study

Target gene	Primer	Oligonucleotide sequences (5' – 3')	Product length (bp)	References
*cdd3*	Time6 Struppi6	TCCAATATAATAAATTAGCATTCC GGCTATTACACGTAATCCAGATA	622	18
*tcdA*	TA1 TA2	ATGATAAGGCAACTTCAGTGG TAAGTTCCTCCTGCTCCATCAA	624	18
*tcdB*	TB1 TB2	GACCTGCTTCAATTGGAGAGA GTAACCTACTT CATAACACCAG	412	18
*cdtA*	CdtA	TGAACCTGGAAAAGGTGATG AGGATTATTTACTGGACCATTTG	375	20
*cdtB*	CdtB	CTTAATGCAAGTAAATACTGAG AACGGATCTCTTGCTTCAGTC	510	20

**Table 2:** Demographic and clinical characteristics of the study population among 144 toxigenic *C. dificile* strain

Patient characteristics	Toxigenic *C. dificile *(n=144)	*tcdA* ^+^ *B* ^+^ (n=124)	*tcdA* ^-^ *B* ^+^ (n=9)	*cdt+* (n=21)	*cdtA* ^+^ *B* ^+^ (n=8)	*cdtA* ^+^ *B* ^-^ (n=4)	*cdtA* ^-^ *B* ^+^ (n=9)
Age (years)							
1-5 6-30 31-50 51-70 70-10	22 (15.3) 36 (25) 25 (17.3) 31 (21.5) 30 (20.9)	20 (16.1) 31 (25) 20 (16.1) 27 (21.8) 26 (21)	0 3 (33.4) 2 (22.2) 3 (33.4) 1 (11)	5 (23.8) 6 (28.5) 4 (19.1) 2 (9.5) 4 (19.1)	2 (25) 3 (37.5) 2 (25) 1 (12.5) 0	2 (50) 1 (25) 0 0 1 (25)	1 (11.2) 2 (22.2) 2 (22.2) 1 (11.2) 3 (33.2)
Gender							
Female Male	73 (50.7) 71 (49.3)	63 (50.8) 61 (49.1)	6 (66.7) 3 (33.3)	8 (38) 13 (62)	3 (37.5) 5 (62.5)	2 (50) 2 (50)	3 (33.3) 6 (66.7)
Defecation (times/day)							
3-5 5-8 >10	73 (50.7) 41 (28.5) 30 (20.8)	64 (51.7) 35 (28.2) 25 (20.1)	5 (55.6) 2 (22.2) 2 (22.2)	14 (66.7) 1 (4.8) 6 (28.5)	5 (62.5) 0 3 (37.5)	3 (75) 0 1 (25)	6 (66.7) 1 (11.1) 2 (22.2)
Antibiotic usage							
Yes No	121 (84) 23 (16)	104 (83.9) 20 (16.1)	8 (88.9) 1(11.1)	18 (85.7) 3 (14.3)	6 (75) 2 (25)	4 (100) 0	9 (100) 0
Underlying diseases							
Cancer Renal failure Dementia Immunodeficiency disorder Diabetes mellitus (DM) Mall nutrition Epilepsy/Allergy	14 (9.8) 7 (4.9) 1 (0.7) 2 (1.4) 4 (2.8) 2 (1.4) 1 (0.7) 1 (0.7)	11 (8.9) 0 1 (0.8) 1 (0.8) 4 (3.22) 2 (1.6) 1 (0.8) 1 (0.8)	1 (11.1) 0 0 0 0 0 0 0	3(14.3) 2 (9.5) 0 0 1 (4.8) 0 0 0	0 1 (12.5) 0 0 1 (12.5) 0 0 0	1 (25) 0 0 0 0 0 0 0	2 (22.2) 1 (11.1) 0 0 0 0 0 0
Hospital wards							
Internal Infectious Surgery Intensive care unit (ICU) Pediatric Oncology Coronary care unit (CCU) Gynecology Gastroenterology Urology Nephrology Endocrinology Orthopedic Psychology Outpatient	32 (22.1) 38 (26.4) 16 (11.1) 21 (14.6) 3 (2) 15 (10.4) 1(0.7) 3 (2) 8 (5.6) 3 (2) 0 1(0.7) 1(0.7) 1(0.7) 1(0.7)	28 (22.6) 32 (25.8) 14 (11.3) 18 (14.5) 3 (2.4) 13 (10.5) 1 (0.8) 3 (2.4) 6 (4.8) 3 (2.4) 0 1 (0.8) 1 (0.8) 0 1 (0.8)	2 (22.2) 2 (22.2) 0 2 (22.2) 0 1 (11.1) 0 0 1 (11.1) 0 0 0 0 1 (11.1) 0	5 (23.8) 7 (33.3) 4 (19) 2 (9.5) 0 1 (4.8) 0 1 (4.8) 1 (4.8) 0 0 0 0 0 0	0 4(50) 2 (25) 2 (25) 0 0 0 0 0 0 0 0 0 0 0	2 (50) 1 (25) 1 (25) 0 0 0 0 0 0 0 0 0 0 0 0	4 (44.4) 2 (22.2) 1 (11.1) 1 (11.1) 0 0 0 1 (11.1) 0 0 0 0 0 0 0

**Table 3:** Distribution of *tcdAB* toxin genes in relation to *cdtAB *binary toxin profiles in 169 clinical strains of *C. difficile*

Toxin profile	Binary toxin (%)				Total
	*cdtA* ^+^ *B* ^+^ (n=8)	*cdtA* ^+^ *B* ^-^ (n=4)	*cdtA* ^-^ *B* ^+^ (n=9)	*cdtA* ^-^ *B* ^-^ (n=148)	
*tcdA* ^+^ *B* ^+ ^	6 (75)	2 (50)	6 (66.7)	110 (74.3)	124 (73.3)
*tcdA* ^+^ *B* ^- ^	1 (12.5)	1 (25)	1 (11.1)	8 (5.4)	11 (6.5)
*tcdA* ^-^ *B* ^+ ^	0	1 (25)	0	8 (5.4)	9 (5.3)
*tcdA* ^-^ *B* ^- ^	1 (12.5)	0	2 (22.2)	22 (14.8)	25 (14.7)

## Results

**Prevalence of CDI**

This study investigated 930 suspected patients to CDI, including 466 males and 464 females. The CDI was confirmed in 169 patients (18.2%), 85 men and 84 women ranged between <1 to 50 years old. The prevalence of CDI varied among the patients in different wards of hospital and was as follow; 22.6% (38/169) in infectious diseases ward, 18.9% (32/169) in internal medicine ward, 12.4% (21/169) in intensive care unit (ICU), 9.5% (16/169) in surgical ward, and 4.8% (8/169) in gastroenterology ward. The lowest prevalence was detected among the patients in bone marrow and kidney transplantation units with less than 5%. Most of the CDI patients presented a defecation rate of 3 times per day, which was higher than CDI-negative patients with ≤2 times per day.

**Frequency of** ***tcdA,*** ***tcdB* and binary toxins among the *C. difficile* strains**

Of total patients with CDI, toxigenic and nontoxigenic* C. difficile* strains were characterized in 85.2% (144/169) and 14.8% (25/169) of the patients, respectively. Toxin genotyping of the strains for* tcdA* and *tcdB *genes revealed the toxin profiles of A^+^B^+^, A^+^B^-^, A^-^B^+^ accounting for 86.1% (124/144), 7.6% (11/144), 6.2% (9/144) among the strains, respectively. Nearly, half of the toxigenic *C. difficile* strains (51.7%) with toxin profile of *tcdA*^+^*B*^+^ were detected among the patients who had 3-5 defecations per day. More than half of the patients (63.6%) with toxin profile of *tcdA*^+^*B*^-^ were seen among the males, while most of the females (66.7%) had toxin profile of *tcdA*^-^*B*^+^. Among all toxigenic patients, cancer was observed as the most common underlying disease. The highest (26.3%) prevalence of toxigenic *C. difficile* strains was observed in patients confined in infectious diseases ward, and the lowest (0.7%) was seen in coronary care unit (CCU), endocrinology and orthopedic wards. Totally, 12.4% (21/169) of the *C. difficile* strains were binary toxin-positive. Among them, *cdtA*^-^*B*^+^,* cdtA*^+^*‏B‏*^+^ and *cdtA*^+^*B*^-^ were detected in 43% (9/21), 38% (8/21) and 19% (4/21) of the strains, respectively. Strains with *cdtA*^+^*‏B‏*^+^ and *cdtA*^-^*B*^+^ profiles were mostly seen among males, while *cdtA*^+^*B*^-^ were equally detected within males and females. The demographic and clinical characteristics of the study population among 144 toxigenic *C. difficile* strains are presented in Table 2.

**Distribution of** ***tcdAB*** **genes in relation to binary toxin profiles**

Most of the* C. difficile* strains with *cdtA*^+^*‏B‏*^+^ and *cdtA*^-^*B*^+^ profiles were found to be *tcdA*^+^*B*^+^. Moreover, half of the strains with *cdtA*^+^*B*^-^ profile were found to be *tcdA*^+^*B*^+^. Interestingly, 12% (3/25) of nontoxigenic* C. difficile* strains (*tcdA*^-^*B*^-^) were found to have either *cdtA*^+^*‏B‏*^+^ or *cdtA*^-^*B*^+^ profiles. None of the strains with *cdtA*^+^*‏B‏*^+^ and *cdtA*^-^*B*^+^ profiles were detected to be *tcdA*^-^*B*^+^. In addition, strains with *cdtA*^+^*‏B‏*^-^ profile carried at least one of the *tcd*A or/and *tcdB* genes. Distribution of *tcdAB* toxin genes in relation to *cdtAB* binary toxin profiles were shown in Table 3.

## Discussion

The structure of the *C. difficile* binary toxin is well known, but few data are available to show the role of this toxin in the pathogenesis of gastrointestinal disease. Currently, there is little information about the clinical relevance and pathogenic role of the ADP-ribosylating toxin CDT in *C. difficile* infections (21). *C. difficile* induces a wide range of diseases from mild diarrhea to PMC, toxic megacolon, and even fulminant colitis. It has been reported that variations in the expression levels of *tcdA* and *tcdB* as the major *C. difficile* toxins cannot account for the wide spectrum of clinical presentations (7, 21). Borriello *et al*. reported no correlation between virulence in a hamster model of antibiotic association colitis and the production of *tcdA* and *tcdB* in vitro (22). Therefore, it was hypothesized that CDT produced by some *C. difficile* strains may be responsible for these manifestations. CDT is a potent cytotoxin, which impairs the functions of mucosal barrier and subsequently can facilitate the action of typical *Clostridial* cytotoxins (23). CDT may also act in synergy with other toxins, depolymerizing the actin cytoskeleton by a complementary mechanism (24). In *C. difficile* strain CD196 that can cause a severe PMC, the production of this additional toxin exacerbates the symptoms of PMC (23). In the present study, *C. difficile* was detected in 169/930 (18.2%) fecal samples collected from diarrheal patients, in which 85.2% (144/169) and 14.8% (25/169) isolates were toxigenic and nontoxigenic, respectively. In our study, the prevalence of toxin profiles including A^+^B^+^, A^+^B^-^, A^-^B^+^ were 86.1% (124/144), 7.6% (11/144), 6.2% (9/144), respectively. In another report by Huang *et al*., the frequency of A^+^B^+^and A^–^B^+^ toxin profiles were 43/71 (60.5%) and 13/71 (18.3%) among patients with recurrent CDI (25). Even though toxigenic *C. difficile* (*tcdA*^+^*B*^+^) plays a major role in the pathogenesis of CDI, several studies represented that CDI caused by A^-^B^+^ strains is increasing significantly (10, 26, 27). Another study performed in Europe demonstrated that 6.2% of *C. difficile* isolates had A^−^B^+^ profile (28). Brazier *et al. *(29) in UK and Pituch *et al.* in Poland (11), reported the isolation rates of 3% and 11% for A^-^B^+^ types, respectively.

In this study, we found that 21 (12.4%) strains of *C. difficile* harbored binary toxin genes. It is noted that 12% (3/25) of our nontoxigenic strains (*tcdA*^-^*B*^-^) were *cdtA*^+^*‏B‏*^+^ and *cdtA*^-^*B*^+^. Although many investigations have been conducted on A^−^B^−^ CDT^+^ toxin profile of* C. difficile* in humans and animals (30-33), the prevalence of this toxin type has been reported to be less than 5% in humans (34). Binary toxin producing strains of *C. difficile* has been reported in recent years, and the prevalence of binary toxin genes vary in different geographic regions worldwide. The deduced prevalence of binary toxin genes in toxigenic strains is high and differences in the findings of prevalence reports may be partly due to the differences in patient (symptomatic or not, adult or children) or strain selection. In addition, as was shown recently by Rupnik *et al.*, the distribution of binary toxin positive strains can vary between hospitals (35). Eckert *et al*. (31) in 2015 reported a range of 17 to 23% for binary toxin prevalence among CDI patients. Another study carried out in UK, revealed that 6.4% of the toxigenic isolates of *C. difficile* possessed the *cdtA* and *cdtB* genes (3). Rupnik *et al.* (36) in 2003, showed a prevalence of 1.6% for the binary toxin in 310 isolates from various hospitals in Japan, Korea and from healthy infants from Indonesia. During 2008 and 2009 Katie *et al.* reported a prevalence 32.5 % of the binary toxin gene (*cdtB*) in 150 consecutive patients with CDI in two hospitals in Ireland (37). In our study, the frequency of A^+^B^+^CDT^+^, A^+^B^+^CDT^-^ andA^-^B^+^CDT^-^ were 6/169 (3.6%), 110/169 (65%), and 8/169 (4.7%), respectively. In a study conducted in Japan the prevalence of A^+^B^+^CDT^+^, A^+^B^+^CDT^-^ and A^-^B^+^CDT^-^ were 4/71(5.6%), 58/71(81.7%), and 9/71 (12.7%), respectively (38, 39). In conclusion, to our knowledge this is the first report for the prevalence of binary toxin genes in *C. difficile* strains isolated from Iran. Further studies are required to investigate the exact role of binary toxins in the pathogenesis of *C. difficile* particularly in patients with chronic diarrhea among Iranian populations.

## Conflict of interests

The authors declare that they have no conflict of interest.
